# The Application of the Improved Jellyfish Search Algorithm in a Site Selection Model of an Emergency Logistics Distribution Center Considering Time Satisfaction

**DOI:** 10.3390/biomimetics8040349

**Published:** 2023-08-06

**Authors:** Ping Li, Xingqi Fan

**Affiliations:** School of Management Science and Engineering, Anhui University of Technology, Ma’anshan 243032, China

**Keywords:** logistics location, emergency facility siting, time satisfaction, artificial jellyfish search algorithm, optimization

## Abstract

In an emergency situation, fast and efficient logistics and distribution are essential for minimizing the impact of a disaster and for safeguarding property. When selecting a distribution center location, time satisfaction needs to be considered, in addition to the general cost factor. The improved jellyfish search algorithm (CIJS), which simulates the bionics of jellyfish foraging, is applied to solve the problem of an emergency logistics and distribution center site selection model considering time satisfaction. The innovation of the CIJS is mainly reflected in two aspects. First, when initializing the population, the two-level logistic map method is used instead of the original logistic map method to improve the diversity and uniform distribution of the population. Second, in the jellyfish search process, a Cauchy strategy is introduced to determine the moving distance of internal motions, which improves the global search capability and prevents the search from falling into local optimal solutions. The superiority of the improved algorithm was verified by testing 20 benchmark functions and applying them to site selection problems of different dimensions. The performance of the CIJS was compared to that of heuristic algorithms through the iterative convergence graph of the algorithm. The experimental results show that the CIJS has higher solution accuracy and faster solution speed than PSO, the WOA, and JS.

## 1. Introduction

### 1.1. Logistics Development and Site Selection Study

With the continuous improvement of people’s living standards, the modern logistics industry has emerged as a vital sector in the national economy. In recent years, there have been numerous studies on logistics systems or supply chains, among which the famous ones are research on supply chain traceability [1,2] and in-depth research on the logistics of the Internet of Things [3], and research on green and sustainable logistics [4] has also gradually entered into scholars’ research horizons. In the supply chain, distribution is one of the important links. Similarly, as an important node in the logistics system, the distribution center is the link between the supply point and the end user, which has an important impact on the overall operation quality and efficiency of the logistics system. A reasonable choice of distribution center location can help enterprises to better control transportation costs, reduce logistics and distribution time, and improve distribution efficiency; thus, the site selection design and optimization scheme of distribution centers are particularly important.

Siting problems are an important branch of transportation problems in operations research and have received wide attention from scholars at home and abroad due to their scientific importance. The location of distribution centers is the optimization process of selecting a certain number of locations to set up a distribution center in a range with several supply points and several demand points [5]. The earliest research on this can be traced back to the Webber problem proposed in 1909: the problem achieves the goal of the shortest total transportation distance between the warehouse and customer by studying the problem of a single warehouse location on the plane [6], which is the beginning of the early siting problem. In the 21st century, various scholars have studied the distribution center location problem by using various traditional methods such as the center-of-gravity method [7,8,9,10] and the analytic hierarchy process (AHP) [11,12,13,14]. However, traditional methods often have disadvantages such as large computational volume and low efficiency, which can bring some difficulties to the solution results. With the increase in the number of variables and constraints in research on siting problems, various new heuristics, such as particle swarm optimization (PSO), grey wolf optimization (GWO), the whale optimization algorithm (WOA), etc. [15,16,17,18,19], have been proposed by different scholars in recent years to solve the siting problem and conduct simulation studies. Although these heuristic algorithms have been well validated and applied in problem-solving, they often have disadvantages such as premature local convergence and poor robustness, which may lead to results with poor accuracy when solving large data models.

For this Np-hard problem, this paper utilizes the artificial jellyfish search algorithm (JS) which has good optimization performance to solve the model. JS is an excellent bionic algorithm based on the feeding behavior of jellyfish, which was proposed by Chou et al. in 2021 [20] and has been applied to solve various environmental problems such as soil structure modeling [21,22,23]. JS has the advantages of having only a few parameters and wide applicability, but it is prone to becoming trapped in local optima when solving large-scale models, leading to low precision of the solution. This paper aims to conduct an in-depth study on improving the population initialization method and the active motion search step size of JS, with the goal of enhancing the diversity of the population and the accuracy of the algorithm and strengthening the algorithm’s search ability beyond the local optima. The superiority of the improved algorithm will be verified through benchmark functions and algorithmic simulations.

### 1.2. Location of Emergency Facilities and Emergency Distribution Centers Study

With the continuous development of society, the occurrence of various emergencies (such as traffic accidents, natural disasters, etc.) may cause a distribution center to be unable to complete its assigned delivery task on time, thus affecting the normal operation of the logistics system [24]. In order to cope with such problems, such as ensuring the efficient transportation of emergency supplies to the battlefield in wartime [25] or ensuring the rapid delivery of disaster relief supplies to disaster areas [26], the key to such problems lies in the reasonable site selection and construction of emergency logistics distribution centers. The construction of emergency logistics distribution centers can not only shorten response and transportation times and reduce construction and operation costs, but it can also improve the emergency security capacity of the logistics system and promote economic prosperity and social stability [27]. The authors of [28] address the siting problem of an urban emergency logistics center, use microblogging big data to obtain data to carry out a risk assessment, establish an emergency logistics positioning model, and use the NSGA-III algorithm to solve and evaluate the siting scheme, and the experimental conclusions can provide empirical references for the city under study to cope with disasters due to rainstorms and floods. The authors of [29] take the emerging coronavirus pneumonia epidemic as the research background, establish a multi-objective mathematical model with the highest vehicle utilization rate and the lowest transportation costs for the study of the emergency material distribution problem, and propose a hybrid multi-inverse optimization algorithm for the experimental simulation, and the computational results provide a solution for the optimization of the distribution of emergency materials via vehicles to cope with the sudden outbreak of the epidemic. In addition, similar to the logistics distribution center of emergency facilities, the siting problem is also reflected in the subway fire emergency station siting, chemical park emergency supplies warehouse siting, and so on [30,31,32,33,34].

Compared with the ordinary site selection problem, the model of the emergency site selection problem is mainly characterized by the consideration of uncertainty conditions or the occurrence of random events and other factors such as constraints. The authors of [35] study the impact of random price factors on cost changes and establish an extended model under the condition of cost uncertainty, and the solution results are favorable to making good choices for the supplier location in the supply chain. There are also many scholars in the field of demand uncertainty who have worked to establish a site selection model and to solve it to verify the reliability of the model [36,37,38]. In addition, some scholars have quantified qualitative factors such as customer satisfaction or overall satisfaction and integrated them into the model to consider the various constraints that may have an impact, and the results of these studies have provided new program references for the location of emergency facilities [39,40]. However, regarding the direction of the consideration of time factor constraints, at this stage, there are a number of studies that consider the time window of the siting problem [41,42], but fewer studies have introduced time satisfaction into the siting of emergency logistics and distribution centers. In this paper, we will introduce time satisfaction to establish a target-planning model based on the consideration of cost uncertainty in the problem of siting emergency facilities, so as to rationally allocate resources and improve the reliability and risk resistance of emergency facilities.

## 2. Model Design

### 2.1. Model Assumptions

**Figure 1:**
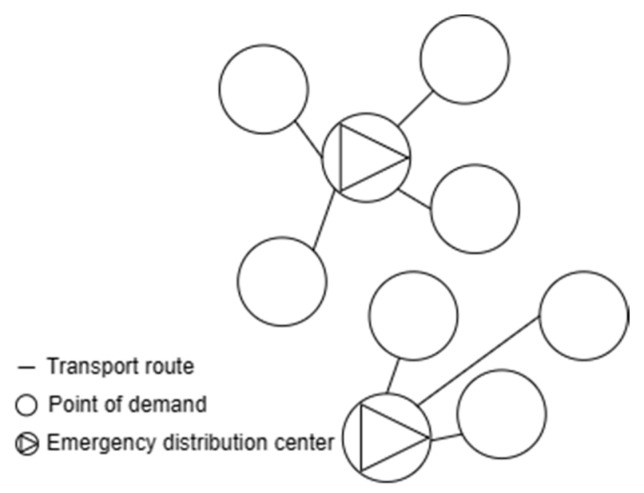
Distribution Network Style.

There are often emergency transportation situations in logistics and the demand for emergency materials is increasing, and emergency distribution centers play an important role in providing timely and effective services. The reasonableness of the location of an emergency distribution center directly affects the rapid response and timely distribution of emergency materials. As shown in Figure 1, the location of an emergency distribution center is based on the original demand points in the region, focusing on transportation costs and time satisfaction factors to select several of them as emergency distribution centers, which will provide follow-up transportation services for other demand points, aiming to ensure logistics transportation after emergencies arise. The logistics nodes set up for efficiency stability, based on the above problem in the model construction, made the following assumptions:

1. All transported materials are of a single type and the volume of goods is measured in weight;
2. The transportation volume is proportional to the transportation cost, and the transportation rate is certain and known;
3. A distribution center can serve multiple demand points, and a single demand point is served by only one distribution center;
4. The quantity demanded at each demand point is certain and known;
5. The materials at each demand point are transported all at once, and the load of the distribution center transportation vehicles can meet all the demands of the responsible demand point.

### 2.2. Model Components

#### 2.2.1. Fixed Costs Component

Fixed costs mainly include construction costs for expanding or rebuilding the original demand point as an emergency distribution center, as well as the daily maintenance costs of the emergency distribution center and other fixed costs.

Using *C_j_* to denote the fixed cost of distribution center *j*, the fixed cost incurred by setting up emergency distribution center *j* is expressed as follows:

$$F_{1}=\sum_{j=1}^{n}C_{j}x_{ij}$$

where *x_ij_* is a 0–1 variable, and, when *x_ij_* = 1, this indicates that *j* is selected as the emergency distribution center responsible for emergency transportation at demand point *I*; otherwise, *x_ij_* = 0.

#### 2.2.2. Variable Cost Component

The variable cost is mainly the transportation cost generated from the emergency distribution center to the demand point, and the transportation cost is mainly calculated by using the transportation rate, transportation distance, and transportation volume; then, the transportation cost from distribution center *j* to demand point *i* is expressed as follows:

$$F_{2}=\sum_{i=1}^{m}\sum_{j=1}^{n}\alpha X_{i}d_{ij}x_{ij}$$

where *α* is the transportation rate, *X_i_* denotes the demand at demand point *i*, and *d_ij_* denotes the distance of demand point *i* from the nearest distribution center *j*.

#### 2.2.3. Time Satisfaction

The biggest difference between an emergency distribution center and an ordinary distribution center is the efficiency of logistics transportation. Since emergency distribution centers need to organize logistics transportation in a short time to cope with emergencies, their operation efficiency is required to be higher. Therefore, this paper reflects the models’ transportation efficiency from the perspective of transportation time between different demand points by constructing a time satisfaction function. Based on the above considerations, this paper introduces a uniform distribution to construct the satisfaction function as shown below:

$$F_{3}=\sum_{i=1}^{m}\sum_{j=1}^{n}X_{i}f(t_{ij})x_{ij}$$

$$f(t_{ij})=\left\{\begin{array}{c} 1,t_{ij}\leq L\\ \frac{U-t_{ij}}{U-L},L<t_{ij}<U\\ 0,t_{ij}\geq U \end{array}\right.$$

$$U-t_{ij}=\frac{d_{2}-d_{ij}}{v},U-L=\frac{d_{2}-d_{1}}{v}$$

where *f*(*t_ij_*) is a uniform distribution function, *d*_2_ is the transport distance in the unsatisfactory case, *d_1_* is the transport distance in the satisfactory case, *d_ij_* is the transport distance from demand point *i* to distribution center *j*, and *v* is the transport speed.

### 2.3. Model Construction

With the above considerations, the objective function of the emergency distribution center site selection model is constructed as follows:

$$\min f\left(F_{1},F_{2},F_{3}\right)=w\cdot \left(\min F_{1}+\min F_{2}\right)+\left(1-w\right)\cdot \max F_{3}$$

i.e.,

(1) $$\min f\left(F_{1},F_{2},F_{3}\right)=w\cdot \left(F_{1}+F_{2}\right)+\left(1-w\right)\cdot F_{3}^{-1}$$

The constraints are as follows:

(2) $$\sum_{i=1}^{m}X_{i}\leq M_{j}$$

(3) $$X_{i}\geq N_{j}$$

(4) $$X_{i}\geq 0,d_{ij}\geq 0,z_{ij}\leq x_{j}$$

Equation (1) is the minimization of the objective function, where *w* denotes the weighting factor and 0 ≤ *w* ≤ 1.

Equation (2) indicates that the sum of the demands of the demand points served by each distribution center must not exceed the maximum capacity of that distribution center.

Equation (3) indicates that the capacity of distribution center *j* should meet the demand of demand point *i* served.

Equation (4) indicates that each demand point corresponds to a unique emergency distribution center and restricts the variables to the delimited area.

## 3. Algorithm Design

### 3.1. Standard Artificial Jellyfish Search Algorithm

Jellyfish live in water at different depths and temperatures around the world. They can move on their own, but in most cases they rely on ocean currents and tides to move. When favorable conditions arise, jellyfish gather in swarms and form jellyfish tides. This phenomenon is caused by factors such as ocean currents, available nutrients, oxygen availability, predation, and temperature, with ocean currents being the main factor. The jellyfish’s own movement and the movement of ocean currents contribute to the formation of jellyfish tides, and the amount of food varies from place to place where the jellyfish go, so the best location is determined by the food ratio.

#### 3.1.1. Initializing the Population

Low initial population diversity may lead to slow convergence or cause the model to easily fall into local optima. The standard jellyfish search algorithm uses the logistic map, which is more convenient to apply and more effective in randomization, to improve population diversity, and this method is simpler and easier to operate than methods such as the Gauss map and Chebyshev map.

The expression for the logistic map is as follows:

$$X_{i+1}=\eta X_{i}(1-X_{i}),0\leq X_{i}\leq 1,\eta =4$$

where *X_i_* is the logistic chaos map value of the *i*-th jellyfish position and η is a control parameter. The randomness of this chaotic sequence was verified in the literature [20] and found to be better when taking the value of (3.99, 4]; here, the value of η is set to 4.

#### 3.1.2. Time Control Mechanism

Ocean currents are rich in nutrients and, therefore, attract a large number of jellyfish. Over time, these jellyfish gather together to form a jellyfish swarm. When the temperature or wind in the current changes, the jellyfish in the swarm will move to another current to form a new jellyfish swarm. The motions in a jellyfish swarm are divided into passive and active motions. At first, jellyfish mainly engage in passive motions, but they tend to move more actively as time goes by. Therefore, the algorithm needs to introduce a temporal control mechanism to handle this situation. The temporal control mechanism controls the transition between ocean currents and intra-population motions using temporal control functions *C*(*t*) and *C*_0_, which are random values that fluctuate from 0 to 1 with time. The formula is as follows:

$$C(t)=\left|\left(1-\frac{t}{M_{i}})\times (2\times rand(0,1)-1\right)\right|$$

where the initial time *C*_0_ = 0.5. If *C*(*t*) > *C*_0_, the movement follows the ocean current, and vice versa for intra-cluster movement.

#### 3.1.3. Simulation of Ocean Currents

Jellyfish either follow currents or move within the community. Ocean currents contain large amounts of nutrients, so jellyfish are attracted to them. The direction of the current (*t*) is determined by the average of all vectors of each jellyfish in the ocean toward the jellyfish currently in the best position, with the following equation:

$$t=\frac{\sum t_{i}}{n_{p}}$$

$$t=X^{*}-df$$

$$df=e_{c}u$$

where *n_p_* is the number of jellyfish, X^*^ is the optimal position of the current jellyfish population, *u* is the average position of all jellyfish, and *df* is the difference between the optimal position of the current jellyfish and the average position of all jellyfish, for *e_c_* controls the factor of attraction, and the equation that determines the difference between the optimal position and the average position of all jellyfish is as follows:

$$e_{c}=\beta \times rand(0,1)$$

df=β×rand(0,1)×u

Thus, the new position of each jellyfish is as follows:

(5) $$X_{i}(t+1)=X_{i}(t)+rand(0,1)\times (X^{*}-\beta \times rand(0,1)\times u),\beta =3$$

where β is a distribution coefficient. After evaluating the validity of the coefficient β associated with the spatial distribution, the authors of [20] took the value of β to be [0.5 10] and found that the best optimal value can be achieved when the value is taken to be 3. In this paper, β = 3.

#### 3.1.4. Simulation of Jellyfish Swarms

The movement of jellyfish in the population is divided into two types: active and passive movement. The mode of locomotion regarding the movement of jellyfish within the population can be expressed as (1 − *C*(*t*)). When *rand*(0, 1) > (1 − *C*(*t*)), the jellyfish population exhibits passive movement. Over time, (1-*C*(*t*)) increases from 0 to 1, so that eventually (1 − *C*(*t*)) > *rand*(0, 1) occurs with a higher probability, and, thus, the jellyfish tend to change from a passive to an active mode of movement when moving internally [20].

When the population starts to form, most jellyfish exhibit passive movements with the following equation:

(6) $$X_{i}(t+1)=X_{i}(t)+\gamma \times rand(0,1)\times (t_{b}-b_{b}),\gamma =0.1$$

where *t_b_* and *b_b_* are the upper and lower bounds of the delimited area, respectively, and γ is a motion coefficient. Similarly, the algorithm works best when the value of γ obtained experimentally after setting the value of γ to [0.05 1] is equal to 0.1 [20].

When a jellyfish starts to make active movements in the group, to simulate this movement, an uninterested jellyfish b is randomly selected and the direction of movement is determined using a vector from the interested jellyfish a to jellyfish b. If the amount of food available to jellyfish b is greater than the amount of food available to jellyfish a of interest, jellyfish a moves toward jellyfish b. If the amount of food available to jellyfish b is less than the amount of food available to jellyfish a of interest, jellyfish a moves away from jellyfish b. Thus, each jellyfish moves in a more favorable direction to find food in the group. The direction of movement and the updated position of the jellyfish are as follows:

$$\begin{array}{c} S=rand(0,1)\times D\\ D=\left\{\begin{array}{l} X_{j}(t)-X_{i}(t),f(X_{i})\geq f(X_{j})\\ X_{i}(t)-X_{j}(t),else \end{array}\right.\\ X_{i}(t+1)=X_{i}(t)+S \end{array}$$

where *X_i_* represents the original jellyfish (the jellyfish of interest), *X_j_* represents the randomly selected jellyfish that is not of interest, *S* represents the search step during active movement, and *D* represents the direction of movement.

#### 3.1.5. Boundary Constraints

In reality, the oceans are spread all over the world and resemble a spherical shape. Therefore, jellyfish that move beyond the boundary will move in the opposite direction. The formula is as follows:

$$\begin{array}{c} X_{i,d}^{\prime }=(X_{i,d}-t_{b,d})+b_{b}(d),\;ifX_{i,d}>t_{b,d}\\ X_{i,d}^{\prime }=(X_{i,d}-b_{b,d})+t(d),\;ifX_{i,d}<b_{b,d} \end{array}$$

where *X_i,d_* is the position of the *i*-th jellyfish in the d-dimension, *X*’*_i,d_* is the updated position of the *i*-th jellyfish in the d-dimension, and *t_b,d_* and *b_b,d_* are the upper and lower boundaries of the delimited region, respectively.

### 3.2. Improved Jellyfish Search Algorithm

#### 3.2.1. Two-Level Logistic Map

The logistic map for initializing populations in the artificial jellyfish search algorithm suffers from the problem of convergence after multiple iterations, and this paper adopts the two-level logistic map method proposed in the literature [43] for initializing populations. The mapping trajectory of the two-level logistic map is jointly determined by two initial values and is more selective. The improved method has a larger selection range of fractal coefficients after Lyapunov exponential analysis, which has the advantages of more selectivity, more uniform distribution, and better chaos. The expression of the two-level logistic map is as follows:

(7) $$\begin{array}{c} X_{i+2}=rX_{i+1}(1-X_{i+1})+(4-r)X_{i}(1-X_{i}),\\ 0\leq X_{i}\leq 1,0\leq r\leq 4 \end{array}$$

where the chaotic mapping value of the updated position of the *i*-th jellyfish is determined by *X_i_* and *X_i+1_*, and r is a fractal coefficient. It has been proven experimentally in the literature [43] that, in the range of [0, 1.2849) and (3.4776, 4], the system is in an obviously chaotic state, except for some individual points which are not in a chaotic state. In addition, after the class randomness test, it was further found that, when r = 0.01, the mapping effect was the most superior, and the number of chaotic sequences that can be selected was also relatively large, so this paper will take the value of r as 0.01.

#### 3.2.2. Adaptive Step Size

The artificial jellyfish search algorithm focused on intra-population movement has specified the way to determine the movement direction, but the intra-population movement distance *Step* has a strong randomness; only the position of the reference jellyfish is considered when simulating the movement, but the superiority of the quality of this jellyfish position is not guaranteed. In this paper, we introduce the *Cauchy* strategy to optimize the reference jellyfish position and the following step to improve the convergence speed and accuracy. The simulated jellyfish *i* is searched outward from the center, and the search area is circular and obeys the *Cauchy* distribution. The new jellyfish positions are updated after adding the *Cauchy* strategy as follows, where *cauchy*(0,1) is the standard *Cauchy* distribution and *f*(*x*) is the one-dimensional standard *Cauchy* stepwise probability density function.

(8) $$\begin{array}{l} X_{i}(t+1)=X_{i}(t)+cauchy(0,1)\cdot D\\ f(x)=\frac{1}{\pi }(\frac{1}{x^{2}+1}),\;-\infty <x<+\infty \end{array}$$

The *Cauchy* distribution has a smooth distribution curve, and its search range is larger, which can effectively jump out of the influence of local search problems in the process.

In summary, the specific steps of the improved jellyfish search algorithm (CIJS) are as follows:

1. Initializing jellyfish populations;
2. Evaluating the fitness value to determine the initial optimal position;
3. Updating the time control parameter *C*(*t*);
4. Updating the jellyfish positions based on ocean currents;
5. Updating the type of movement and updating the position of the jellyfish for types a and b, respectively;
6. Re-evaluating the fitness value and updating the jellyfish’s optimal position;
7. Determining whether the maximum number of iterations is satisfied, and, if so, outputting the optimal position and the global optimal solution; otherwise, the algorithm will return to step 3 and re-iterate the calculation.

Based on the above algorithm steps, the CIJS can be described as shown in Algorithm 1.
**Algorithm 1** Pseudo-code of CIJS.**Input:** Evaluation function *f(x)* Number of jellyfish *n_p_* Maximum number of iterations *M_i_* Top and bottom bounds on the value of the d-dimension *t_b,d_* & *b_b,d_***Output:** Optimal fitness value1:**Begin**2:Initializing jellyfish populations *X_i_* using the two-levels logistic map method by Equation (7).3:Calculate the quantity of food for *X_i_* and find the jellyfish locations with the most food X^*^.4: Initialization time *t* = 1.5:  **Repeat**
6:
   **For i = 1: *****n_p_***
** do**
7:   Calculate the time control *C*(*t*).8:   If *C*(*t*) > 0.5, jellyfish follow ocean currents.9:     Updating jellyfish locations by Equation (5)10:   Otherwise, jellyfish moving within populations.11:    If *rand*(0,1) < 1 − *C(t)*, jellyfish adopt passive movements in populations.12:Updating jellyfish locations by Equation (6)13:Otherwise, jellyfish adopt active movements in populations.14:Updating jellyfish locations by Equation (8)15:**End if**16:**End if**17:Check the boundaries.18:  Calculate current quantity of food for *X_i_* and find the jellyfish locations with the most food X^*^.19:**End for i**20:Update the time *t* = *t* + 121:**Until** t > *M_i_* stop22:**End**

## 4. Experimental Simulation and Analysis

### 4.1. Baseline Function Test

In this paper, 20 of the benchmark functions listed in the literature [44] (as shown in Table 1) were selected and compared to verify the effectiveness and superiority of the improved algorithm by solving through the use of particle swarm optimization (PSO), whale optimization algorithm (WOA), the artificial jellyfish search algorithm (JS), and the improved jellyfish search algorithm (CIJS). The first to the sixth functions are single-peaked functions to check the speed and accuracy of the improved algorithm; the seventh to the twentieth functions are multi-peaked functions to check the ability of the improved algorithm to jump out of the local optimum.

The parameter settings of each algorithm are shown in Table 2, and the algorithms were run on a computer with an inter(R) Core i5-9400 processor and the running environment Windows 10, using MatlabR2018b software for 30 iterations each, and these 30 experiments were summarized and analyzed for comparative data (including the mean, optimal fitness value, and standard deviation) as shown in Table 3.

**Figure 2:**
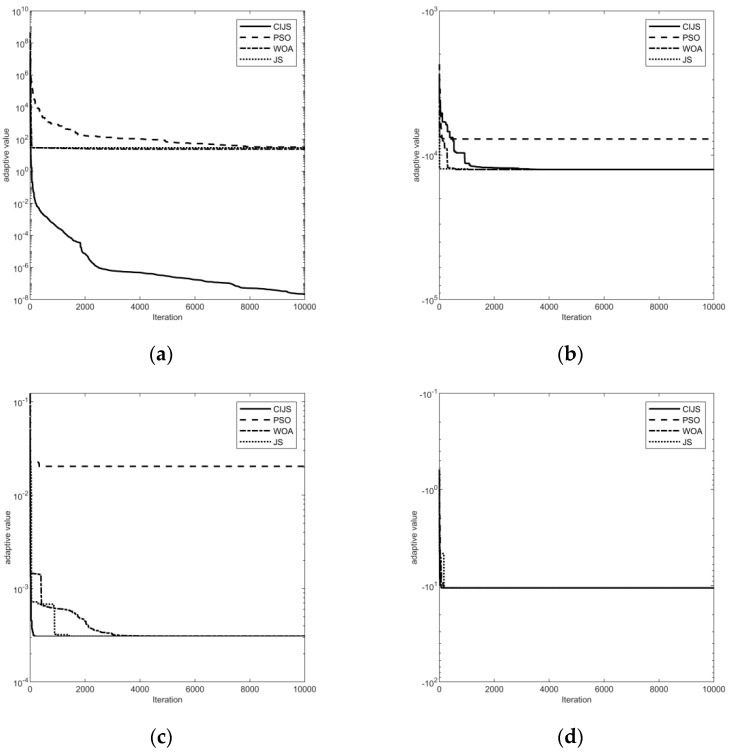
Distribution network style: (**a**) iterative comparison image of the 4th benchmark function; (**b**) iterative comparison image of the 7th benchmark function; (**c**) iterative comparison image of the 13th benchmark function; and (**d**) iterative comparison image of the 20th benchmark function.

According to the comparison of the data in Table 3 and the iterative curves in Figure 2, it can be seen that the CIJS performance is higher than the other three algorithms when dealing with the 1st to 6th single-peaked functions; when dealing with multi-peaked functions, the CIJS used for the 7th function iteration to the theoretical value is slower than the WOA and JS in terms of the number of iterations. Although the CIJS can find the theoretical value of the test function, the 13th function is better than the WOA and JS in terms of stability. In addition, when dealing with the 18th to 20th fixed-dimensional multi-peaked function Shekels, there is almost no difference in solution accuracy and solution speed between the CIJS compared to the JS and WOA, and all three algorithms can iterate better to the theoretical value. The PSO search results in the benchmark function tests were generally poor. Overall, the iteration results obtained via the CIJS with 30 iterations of different test functions are better than the other three algorithms and have certain advantages in solving various benchmark functions, but there is still room for improvement in the iteration speed.

In summary, after selecting 20 benchmark function problems to test the performance of the CIJS, the results of the four algorithms were statistically analyzed, and the improved jellyfish search algorithm proposed in this paper has overall a better optimization seeking ability and iterative effects.

### 4.2. Algorithm Simulation

Experiments were conducted using the improved jellyfish search algorithm for the 30-dimensional logistics center arithmetic and the 100-dimensional logistics center arithmetic, with the parameters set as shown in Table 4.

#### 4.2.1. Site Selection for the 30-Dimensional Emergency Logistics Distribution Center

Here, the co-ordinates of the 30 demand points were collected, and Table 5 shows the co-ordinates and the amount of material to be distributed. The CIJS population size parameter is 50 and the maximum number of iterations is 100, and the optimal results of the solution for the different numbers of distribution centers were selected for 30 iterations in the same computer environment as above, as shown in Table 6.

**Figure 3:**
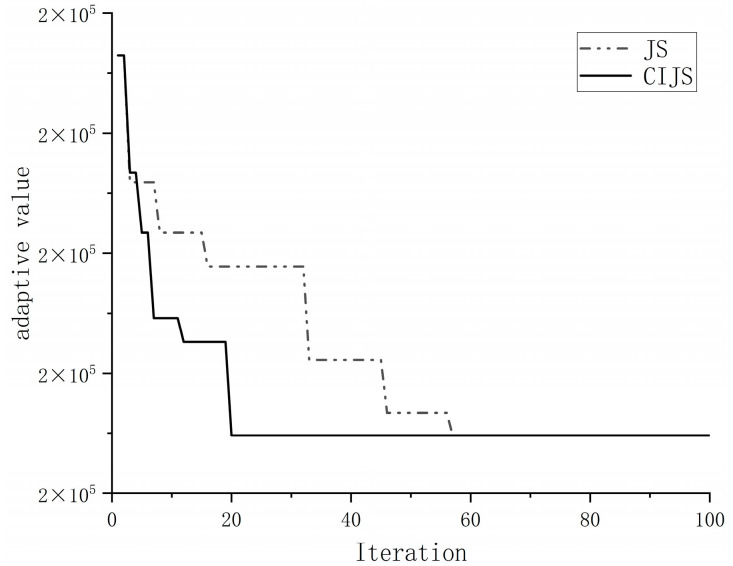
Comparison of iteration curves.

Once again, the population size parameter of the standard artificial jellyfish search algorithm was set to 50, the maximum number of iterations was set to 100, and 30 experiments were performed in the same computer environment. The optimal iteration curves when six emergency logistics distribution centers are selected were compared with the CIJS experimental results, as shown in Figure 3.

The optimal fitness value of both algorithms in 30 trials can reach 184813, but the average time taken to solve the model using the CIJS is 17.65 s and 18.34 s using JS, and the figure shows that the CIJS can iterate the optimal value at the 20th iteration, while JS can only iterate the optimal value after the 50th iteration. Therefore, it can be seen that, even though the accuracy of the improved jellyfish search algorithm in the 30-dimensional example is comparable to that of the JS, the CIJS solves the model faster.

#### 4.2.2. Site Selection for the 100-Dimensional Emergency Logistics Distribution Center

After the above 30-dimensional experiments, the co-ordinates and demand of 100 demand points in higher dimensions as shown in Table 7 were collected for experiments. The CIJS population size parameter was also set to 50, the maximum number of iterations was 500, and the selected distribution center numbers and fitness value obtained by solving for different numbers of distribution centers for 30 iterations in the same computer environment were as above, as shown in Table 8.

**Figure 4:**
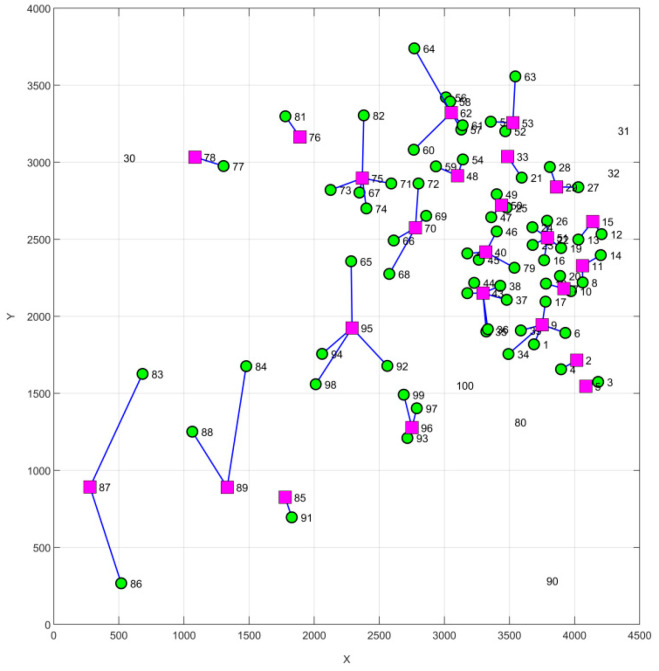
Selected points and transport routes.

The demand points and transportation routes responsible for selecting 30 emergency logistics distribution centers are shown in Figure 4, where the square represents the distribution center, the circle represents the demand point, and the connecting line between the two shapes represents the transportation route.

**Figure 5:**
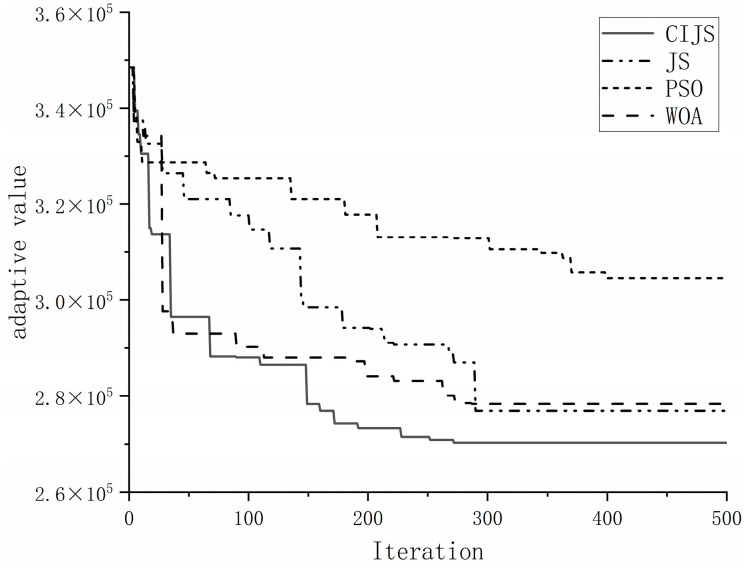
Comparison of iteration curves.

PSO, WOA, and JS were used to perform 30 experiments in the same computer environment with the same parameters as in the benchmark function test above, and the population size was set to 50 and the maximum number of iterations was set to 500. In order to compare the results of the four algorithms more intuitively, a comparison of the optimal iteration curves when 30 emergency logistics and distribution centers are selected is shown in Figure 5. From the figure, it can be seen that the CIJS can iterate to the optimal value in the shortest number of iterations and has the highest solution accuracy. The WOA is completed earlier than the JS iteration, but its accuracy is poorer compared to JS, which may be caught in the local optimum; instead, the final fitness value of the JS iteration is better than that of the WOA. PSO has the slowest search speed and lowest solution accuracy.

Table 9 shows the optimal fitness values, the optimal number of iterations, and the average iteration time of the PSO, WOA, JS, and CIJS in 30 experiments when selecting 30 emergency logistics and distribution centers. From the statistical analysis in the table, it can be seen that, in the 100-dimensional distribution center selection problem, compared with WOA, JS, and PSO, the result of the CIJS solution model is 270,296, and the average iteration time is 217 s, and these two values are optimal in the results of the four algorithms. Thus, it can be shown that the improved jellyfish search algorithm can better ensure that the problem can be solved by jumping out of the local optimum, and the result with the highest accuracy can be produced in the shortest time.

In summary, the performance of the CIJS was further verified by applying the improved bionics algorithm to model solving problems with different dimensions and comparing its computational results with those of PSO, WOA, and JS. The results of the statistical analysis show that the CIJS has a faster iteration speed and higher solution accuracy compared to the other algorithms. The above arithmetic simulation can be a reference for similar objective-planning problems such as logistics site selection.

## 5. Conclusions

In this paper, based on the consideration of logistics and distribution transportation costs, the material transportation time satisfaction index was introduced to establish an emergency logistics distribution center site selection model, which can better respond to the influencing factors of distribution center site selection in emergency situations.

An improved jellyfish search algorithm was used to solve the emergency distribution center siting problem based on the proposed siting model. The standard artificial jellyfish search algorithm simulates jellyfish foraging behavior through the bionic phenomenon, which has better search capabilities. In order to address the problem where JS easily falls into the local optimum when searching, the original way of initializing the population is changed, and the two-level logistic map method is introduced to increase the diversity of the population; at the same time, the Cauchy variation strategy is introduced to make it easier to determine the searching step length in the active searching. The above improvements can accelerate the convergence speed of the algorithm, ensure that the search jumps out of the local optimum, and improve the solution accuracy. The convergence and stability of the algorithm were also analyzed by using the benchmark function test experiments, and the superiority of the CIJS was verified by comparing it to other algorithms. The improved jellyfish search algorithm can find the optimal solution faster, thus improving the quality of the solution.

In the emergency distribution center siting problem, the improved algorithm can find the optimal siting solution faster and improve emergency response efficiency. Meanwhile, simulating the examples of logistics and distribution centers with different dimensions, the improved jellyfish search algorithm is able to adapt to different emergency scenarios, obtain good results, and provide a more reliable siting solution than some other algorithms.

However, there are some limitations to this study. First, for some large-scale and high-dimensional optimization problems, the computational complexity of the CIJS may increase, leading to a decrease in the efficiency of the algorithm. Second, the setting of parameters has a great impact on the performance of the algorithm, and further experiments and analysis are needed to optimize the selection of parameters. Although the CIJS shows potential in solving optimization problems, further research is needed to verify its application value in practical problems.

In addition, the modeling considerations in this paper are limited to time satisfaction and cost issues, ignoring the effects of uncertain events such as supply chain reliability, environmental risks, or some uncertain events such as traffic congestion and bad weather. Even the application of the improved algorithm is only simulated and verified in the site selection problem, which lacks the verification of other real scenarios. Although the simulation analysis in this paper shows that the improved jellyfish search algorithm has some advantages in terms of the solution results and operation in the site selection problem, subsequent verification of new scenarios, such as network optimization and power system scheduling, is needed.

## Figures and Tables

**Table 1:** Basis functions.

Fun. No.	Function	Range	Opt.
1	Sphere	[−100, 100]	0
2	Schwefel 2.22	[−10, 10]	0
3	Schwefel 1.2	[−100, 100]	0
4	Rosenbrock	[−30, 30]	0
5	Step	[−100, 100]	0
6	Quartic	[−1.28, 1.28]	0
7	Schwefel	[−500, 500]	−12,569.5
8	Rastrigin	[−5.12, 5.12]	0
9	Ackley	[−32, 32]	0
10	Griewank	[−600, 600]	0
11	Penalized2	[−50, 50]	0
12	Foxholes	[−65.536, 65.536]	0.998
13	Kowalik	[−5, 5]	0.00031
14	Six-Hump Camel Back	[−5, 5]	−1.03163
15	Goldstein–Price	[−2, 2]	3
16	Hartman3	[0, 1]	−3.86
17	Hartman6	[0, 1]	−3.32
18	Shekel5	[0, 10]	−10.15
19	Shekel7	[0, 10]	−10.4
20	Shekel10	[0, 10]	−10.53

**Table 2:** Parameter setting.

Algorithm	Parameter
PSO	NP = 50, MI = 10,000, personal learning coefficient = 2, global learning coefficient = 2, inertia weight = 0.9
WOA	NP = 50, MI = 10,000, fluctuation range: decreased from 2 to 0, coefficient of the logarithmic spiral shape = 1
JS	NP = 50, MI = 10,000
CIJS	NP = 50, MI = 10,000

**Table 3:** Summary of experimental results.

No.	CIJS			PSO			WOA			JS		
	Mean	Opt.	St.	Mean	Opt.	St.	Mean	Opt.	St.	Mean	Opt.	St.
1	0	0	0	8.70 × 10^−3^	6.58 × 10^−3^	3.12 × 10^−3^	0	0	0	0	0	0
2	0	0	0	2.35	5.34 × 10^−3^	8.03	0	0	0	0	0	0
3	0	0	0	12.78	3.20	2.48	2.54 × 10^−1^	2.53 × 10^−1^	2.69 × 10^−3^	3.92 × 10^−2^	5.70 × 10^−4^	8.34 × 10^−2^
4	0	0	0	1.22 × 10^2^	32.60	71.46	23.69	23.68	4.79 × 10^−2^	28.76	28.72	8.77 × 10^−3^
5	0	0	0	1.37 × 10^−2^	1.32 × 10^−2^	2.21 × 10^−3^	0	0	0	5.68 × 10^−1^	4.04 × 10^−1^	2.40 × 10^−1^
6	0	0	0	1.66 × 10^−3^	1.66 × 10^−3^	0	0	0	0	0	0	0
7	−1.26 × 10^4^	−1.26 × 10^4^	0	−7.22 × 10^3^	−7.59 × 10^3^	3.76 × 10^2^	−1.26 × 10^4^	−1.26 × 10^4^	2.41 × 10^−3^	−1.26 × 10^4^	−1.26 × 10^4^	0
8	0	0	0	50.88	19.10	28.84	0	0	0	0	0	0
9	0	0	0	1.38	2.26 × 10^−1^	5.31 × 10^−1^	0	0	0	0	0	0
10	0	0	0	1.56 × 10^−2^	1.54 × 10^−2^	2.96 × 10^−5^	0	0	0	0	0	0
11	0	0	0	5.02 × 10^−2^	4.91 × 10^−2^	4.31 × 10^−3^	0	0	0	7.30 × 10^−2^	7.06 × 10^−2^	1.30 × 10^−2^
12	9.98 × 10^−1^	9.98 × 10^−1^	0	9.98 × 10^−1^	9.98 × 10^−1^	0	9.98 × 10^−1^	9.98 × 10^−1^	0	9.98 × 10^−1^	9.98 × 10^−1^	0
13	3.10 × 10^−4^	3.10 × 10^−4^	4.00 × 10^−4^	2.25 × 10^−2^	2.04 × 10^−2^	3.93 × 10^−4^	3.07 × 10^−4^	3.07 × 10^−4^	0	3.47 × 10^−4^	3.40 × 10^−4^	4.15 × 10^−5^
14	−1.03	−1.03	0	−1.03	−1.03	0	−1.03	−1.03	0	−1.03	−1.03	0
15	3	3	0	3	3	0	3	3	0	3	3	0
16	−3.86	−3.86	0	−3.86	−3.86	3.11 × 10^−15^	−3.86	−3.86	3.11 × 10^−15^	−3.86	−3.86	3.11 × 10^−15^
17	−3.32	−3.32	0	−3.32	−3.32	0	−3.32	−3.32	0	−3.32	−3.32	0
18	−10.15	−10.15	0	−10.15	−10.15	0	−10.15	−10.15	0	−10.05	−10.05	0
19	−10.4	−10.4	0	−10.4	−10.4	0	−10.4	−10.4	0	−10.4	−10.4	0
20	−10.53	−10.53	0	−10.53	−10.53	8.88 × 10^−15^	−10.53	−10.53	0	−10.53	−10.53	0

**Table 4:** Parameter setting.

Parameter	Parameter Description	Parameter Value	Unit
*v*	Transfer speed	50	km/h
*d*_1_	Satisfactory transfer distance	40	km
*d*_2_	Unsatisfactory transfer distance	80	km
*α*	Transportation rates	0.5	CNY/km·T
*C_j_*	Fixed cost of distribution center	10,000	CNY/piece
*w*	Cost weight	40	%

**Table 5:** Points-of-demand information.

No.	X	Y	Demand (T)	No.	X	Y	Demand (T)
1	1305	2312	20	16	3716	1678	80
2	3638	1315	90	17	3917	2179	90
3	4178	2244	90	18	4060	2370	70
4	3713	1399	60	19	3781	2212	100
5	3489	1535	70	20	3675	2578	50
6	3325	1556	70	21	3430	2838	50
7	3237	1229	40	22	4264	2931	50
8	4195	1044	90	23	3428	1908	80
9	4313	790	90	24	3508	2376	70
10	4385	570	70	25	3395	2643	80
11	3006	1970	60	26	3438	3201	40
12	2563	1756	40	27	2936	3240	40
13	2789	1461	40	28	3141	3550	60
14	2382	1676	40	29	2546	2537	70
15	1331	695	20	30	2779	2826	50

**Table 6:** Site selection options.

Num. of Centers	Selected No.	Fitness Value
3	20,9,6	320,466
4	17,9,25,6	258,882
5	12,21,5,17,9	212,676
6	17,25,12,5,30,9	184,813

**Table 7:** Points-of-demand information.

No.	X	Y	Dem.	No.	X	Y	Dem.	No.	X	Y	Dem.	No.	X	Y	Dem.
1	3688	1818	90	26	3789	2620	80	51	3792	2510	60	76	1890	3164	60
2	4016	1715	100	27	4029	2838	100	52	3468	3201	50	77	1304	2975	30
3	4181	1574	110	28	3810	2969	90	53	3526	3256	60	78	1084	3033	40
4	3896	1656	90	29	3862	2839	80	54	3142	3018	50	79	3538	2315	100
5	4087	1546	100	30	467	3029	90	55	3356	3263	50	80	3470	1313	110
6	3929	1892	90	31	4263	3206	110	56	3012	3421	40	81	1779	3298	30
7	3918	2179	90	32	4186	2931	120	57	3130	3212	40	82	2381	3304	50
8	4062	2220	100	33	3486	3037	110	58	3044	3394	70	83	682	1626	20
9	3751	1945	80	34	3492	1755	50	59	2935	2973	60	84	1478	1676	20
10	3972	2163	90	35	3322	1901	50	60	2765	3081	40	85	1777	825	30
11	4061	2328	100	36	3334	1916	40	61	3140	3240	60	86	518	267	20
12	4207	2533	120	37	3479	2107	50	62	3053	3321	70	87	278	892	20
13	4029	2498	100	38	3429	2198	50	63	3545	3557	70	88	1064	1251	30
14	4201	2397	120	39	3587	1908	60	64	2769	3739	50	89	1332	890	70
15	4139	2615	110	40	3318	2417	40	65	2284	2357	30	90	3715	284	40
16	3766	2364	90	41	3176	2408	30	66	2611	2492	40	91	1828	695	50
17	3777	2095	80	42	3176	2150	30	67	2348	2803	50	92	2562	1678	60
18	3780	2212	80	43	3296	2150	50	68	2577	2275	40	93	2716	1210	50
19	3896	2443	90	44	3229	2217	60	69	2860	2652	50	94	2061	1756	50
20	3888	2262	90	45	3264	2367	70	70	2778	2574	50	95	2291	1924	60
21	3594	2900	100	46	3402	2551	70	71	2592	2862	40	96	2751	1277	70
22	3796	2499	90	47	3360	2643	60	72	2801	2862	50	97	2788	1403	50
23	3678	2463	80	48	3101	2912	80	73	2126	2820	80	98	2012	1559	20
24	3676	2578	70	49	3402	2792	70	74	2401	2700	80	99	2688	1491	50
25	3478	2705	60	50	3439	2721	50	75	2370	2896	70	100	3020	1552	70

**Table 8:** Site selection options.

Num. of Centers	Selected No.	Fitness Value
5	33,67,19,99,1	985,207
10	89,50,38,77,61,4,67,19,99,27	632,321
20	38,62,30,40,51,12,33,70,7,76,89,59,9,80,75,5,95,96,27,49	386,028
30	51,30,62,80,29,33,32,15,90,75,9,76,43,2,70,85,11,89,7,95,31,5,48,53,50,96,87,40,78,100	270,296

**Table 9:** Iterative values.

Algorithm	Opt.	Iterations	Average Iteration Time
CIJS	270,296	272	217
JS	276,902	290	233
WOA	278,335	287	237
PSO	304,577	399	301

## Data Availability

Not applicable.
